# Untargeted Plant Metabolomics: Evaluation of Lyophilization as a Sample Preparation Technique

**DOI:** 10.3390/metabo13060686

**Published:** 2023-05-25

**Authors:** Christina Maisl, Maria Doppler, Bernhard Seidl, Christoph Bueschl, Rainer Schuhmacher

**Affiliations:** 1 Department of Agrobiotechnology IFA-Tulln, Institute of Bioanalytics and Agro-Metabolomics, University of Natural Resources and Life Sciences, Vienna, Konrad-Lorenz-Str. 20, 3430 Tulln, Austria; 2 Core Facility Bioactive Molecules: Screening and Analysis, University of Natural Resources and Life Sciences, Vienna, Konrad-Lorenz-Str. 20, 3430 Tulln, Austria

**Keywords:** freeze-drying, concentration, enrichment, stabilization, RP-LC-HRMS

## Abstract

Lyophilization is a common method used for stabilizing biological samples prior to storage or to concentrate extracts. However, it is possible that this process may alter the metabolic composition or lead to the loss of metabolites. In this study, the performance of lyophilization is investigated in the example of wheat roots. To this end, native and ^13^C-labelled, fresh or already lyophilized root samples, and (diluted) extracts with dilution factors up to 32 and authentic reference standards were investigated. All samples were analyzed using RP-LC-HRMS. Results show that using lyophilization for the stabilization of plant material altered the metabolic sample composition. Overall, 7% of all wheat metabolites detected in non-lyophilized samples were not detected in dried samples anymore, and up to 43% of the remaining metabolites exhibited significantly increased or decreased abundances. With respect to extract concentration, less than 5% of the expected metabolites were completely lost by lyophilization and the recovery rates of the remaining metabolites were slightly reduced with increasing concentration factors to an average of 85% at an enrichment factor of 32. Compound annotation did not indicate specific classes of wheat metabolites to be affected.

## 1. Introduction

Lyophilization, also known as freeze-drying, is widely used in various fields, such as pharmaceutical, cosmeceutical, nutraceutical, chemical, and food industries [1,2]. It can be used for, e.g., the preservation of sensitive materials such as microorganisms, nanoparticulate systems, nucleic acids, and proteins, as well as to improve drug stability [3,4]; to stabilize vaccine formulations [5]; to preserve microorganisms [6]; to pre-process for the storage of serum and plasma [7]; to dry polymer nanoparticles [8], plant extracts [1] or crude plant material [9]; and to preserve food and increase the shelf life of agricultural products [10,11,12].

Lyophilization is also used in metabolomics research. It removes water while retaining bioactive ingredients (e.g., antioxidant compounds) [13]. For example, in plant metabolomics experiments, biological materials are often dried to stabilize them and prevent enzymatic activities and metabolite decomposition [14,15,16]. This technique can also be applied to, e.g., dry fruits for the study of anthocyanins, which are used in food and pharmaceutical industries as natural pigments [17]; dry vegetable samples prior to NMR spectroscopy analysis [15]; prepare urine samples for headspace-SPME-GC-MS analysis of volatile organic compounds [18]; dry fecal samples to preserve microbial and chemical stability, as well as standardize samples by dry weight for gut microbiota studies [19]; and to dry tissue powder for biological and clinical research [20]. Furthermore, lyophilization can be used to reduce sample volume and to concentrate compounds in aqueous samples that would otherwise be below the limit of detection [21], e.g., plant root exudates [22].

During lyophilization, samples are frozen beforehand, and due to reduced pressure, frozen moisture content sublimes out of the sample. Consequently, lyophilization is a mild technique [10]. It also offers advantages in terms of enzyme stability and reproducibility [23] and limits oxidative changes of metabolites due to the applied vacuum [1].

For these reasons, lyophilization is therefore regarded as the golden standard. However, even lyophilization, as with every sample preparation step, bears the risk that it alters the sample composition to a certain extent. For example, studies showed that the extraction of metabolites from wheat leaves using mixtures of methanol can generate artifacts during sample extraction and short-term storage [24]. Lyophilization can also lead to undesirable side effects, e.g., long and variable reconstitution times of lyophilized products containing highly concentrated proteins [25]. Other disadvantages that were reported for lyophilization are the potential loss of volatile compounds that are prone to sublimation [10], decreased metabolic activities of certain bacterial strains and their antibiotic/chemical resistance [6], and various stresses that can lead to chemical and physical instabilities of nanoparticles, vaccines, genes, and microorganisms (e.g., physical alteration caused by the removal of the molecule hydration shell or stresses related to the internal environment of bacteria, such as osmolarity change or ice formation) [3].

There are some studies that investigate specific applications of lyophilization or its effect on certain compounds or compound classes. For example, García-Salas and colleagues investigated the stability of phenolic compounds of lemons in different drying processes and showed that low temperature drying lowered the phenolic content of lemon [26]. Other studies reported that the drying method influences the bioactivity and chemical composition of plant material [27,28,29,30,31,32]. Generally, it appears that depending on the plant species, lyophilization has different effects on metabolite levels, according to Oikawa and colleagues, who investigated *Arabidopsis* plants and pear fruits [33].

Potential alternatives for lyophilization as a technique to remove water are, e.g., oven or microwave drying; however, both are not as mild as lyophilization [10], but are cheaper and faster than lyophilization [10].

To the best of the authors’ knowledge, only few studies examined the impact of lyophilization on the metabolic composition of biological samples [33]. Furthermore, no systematic investigation probed the effect of lyophilization for concentrating extracts. However, it is crucial to evaluate and understand potential alterations of the sample composition to assess the suitability of the lyophilization step and, if necessary, to account for its negative effects during sample preparation. Here, the extent of changes in the metabolic composition introduced by lyophilization for (i) the stabilization of plant material and (ii) concentration of extracts are investigated systematically.

## 2. Materials and Methods

### 2.1. Chemicals, Reference Standards and Plant Material

The frozen U-^13^C-labelled root material of Remus wheat cultivars were prepared as described by Ceranic, et al. (2020) [34], and native Remus plants were kindly provided by Barbara Steiner (University of Natural Resources and Life Sciences, Vienna, Department of Agrobiotechnology IFA-Tulln, Institute of Biotechnology in Plant Production, Konrad-Lorenz-Str. 20, 3430 Tulln, Austria). Details on plant cultivation and sampling can be found in [35] and in Section 2.3.1.

LC-grade Methanol (MeOH) and acetonitrile (ACN) were purchased from Riedel de Haen, Honeywell (LC-MS grade, >99.9% purity, Seelze, Germany). MS-grade formic acid (FA) was purchased from Sigma-Aldrich (Vienna, Austria). ELGA water was obtained from an ELGA Purelab Ultra-AN-MK2 system (Veolia Water; Vienna, Austria).

The following authentic reference standards were purchased and used in this study for the reference standard mix: ethyl ferulate, methyl 3-(4-hydroxyphenyl) acrylate, 3-(4-hydroxyphenyl)propionate, scopoletin, linolenic acid, d-desthiobiotin, 5’-deoxy-5’-(methylthio)adenosine, 5-methoxy-3-indoleacetic acid, chlorogenic acid, L-tryptophan, reserpine (all from Sigma-Aldrich, Vienna, Austria), linoleic acid (TCI, Tokio, Japan), L-phenylalanine (Roth, Karlsruhe, Germany), schaftoside (PhytoLab, Vestenbergsgreuth, Germany), and deoxynivalenol (Romerlabs, Getzersdorf, Austria).

For annotation, ferulic acid was used (Fluka, Vienna, Austria).

### 2.2. Overview Experimental Setup

#### 2.2.1. Experimental Setup

**Figure 1:**
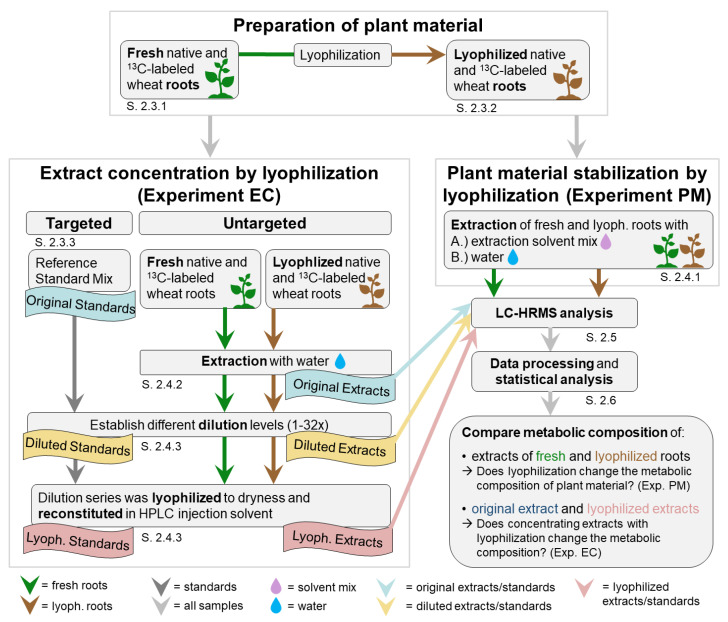
Overview of the experimental setup showing the workflow of both experiments and the section (S. x), where detailed descriptions of the respective step can be found.

Generally, lyophilization is used for a reduction in solvents, which can be used for the enrichment of samples with low-abundant metabolites (referred to as “concentrating”; solvent is removed to increase metabolite concentration), or for the conservation of plant samples (referred to as “stabilization” or “drying”; water is removed to prevent enzymatic activity and decomposition of metabolites). Consequently, two experiments were performed (Figure 1): the first experiment investigated the effect of plant material stabilization by lyophilization (including the reconstitution of the lyophilized sample before subsequent measurement) with respect to the sample’s metabolic composition as well as the influence of extraction solvent (experiment PM), while the second experiment investigated the effect of aqueous sample concentration by lyophilization with respect to the sample’s metabolic composition (experiment EC). For this, fresh and freeze-dried root samples were extracted with water and further diluted with water to have a practicable simulation of samples with low-abundant metabolite concentrations (e.g., root exudate samples). Next, diluted samples were concentrated (lyophilized and reconstituted), and original and concentrated extracts were compared. Furthermore, a mix of authentic reference standards was used as a proof of concept. The extracts, which were diluted, lyophilized, and reconstituted, are referred to as “concentrated extracts”.

#### 2.2.2. Stable Isotopically Labelled Plant Material for Global Internal Standardization

Both experiments used mixtures of extracts of native and globally ^13^C-labelled plant roots. The mixtures of native and ^13^C-labelled root extracts were prepared using an established workflow that allows extracting only pairs of native and ^13^C-labelled metabolite ions, thereby filtering out any unspecific features resulting from background compounds (i.e., non-wheat metabolites such as plasticizers, contaminants of solvent). This allows for focusing the analysis on only the true wheat root metabolites [36,37]. The detailed workflow and protocols for plant cultivation and handling are described in Ceranic, et al. (2020) [34] and in Section 2.3.1.

Both experiments (PM and EC) were carried out with either fresh and dried (native and ^13^C-labelled) plant roots in parallel. For this, native and ^13^C-labelled plants were cultivated, harvested, and frozen separately. Lyophilization (to obtain dried native and labelled roots), milling, and extraction, was performed separately for both fresh and dried either native or ^13^C-labelled roots. Only after extraction, native and ^13^C-labelled extracts were mixed (1 + 1, *v*/*v*; labelled material was added for internal standardization) as explained later. All of these sample preparation steps were performed for both experiments. For experiment EC, the mixed extracts were first diluted (defined dilution factors up to 32), concentrated to dryness by lyophilization, and reconstituted with solvent. Therefore, in experiment EC, also the composition of the labelled material can potentially be changed by the concentration step together with the native constituents, making the labelled material a relative reference, which compensates for metabolic changes by lyophilization.

### 2.3. Sample Preparation

#### 2.3.1. Fresh Root Samples

Native and ^13^C-labelled Remus plants at the flowering stage were used. The handling of the labelled plants is described by Ceranic and colleagues [34]. Briefly summarized: ^13^C labelling of wheat plants was performed in an air-tight growth chamber (phytolabelbox), where environmental conditions were controlled and highly enriched ^13^CO_2_ was used. Seeds were germinated, vernalized, transferred into the phytolabelbox, and cultured as described by Ceranic and colleagues [34]. At the flowering stage, roots were cut off, immediately frozen in liquid nitrogen, and cleaned from growth substrate. Samples were stored at −80 °C.

Native plants were cultivated in the glass house without ^13^CO_2_ and harvested at the flowering stage as described by Ceranic and colleagues [38]. Briefly summarized, the plants were removed from the pot and remaining soil was washed from the roots with water. Cleaned roots were dried with paper towels, cut from the stem, and immediately frozen in liquid nitrogen. Then, roots were stored at −80 °C until further sample preparation. Some of the frozen fresh roots were immediately milled to a fine powder (MM301 Retsch, Haan, Germany; 30 Hz for 60 s), which was also stored at −80 °C until extraction.

#### 2.3.2. Lyophilization of Root Samples

Frozen intact roots of native and labelled plants were lyophilized at −80 °C for 3 days (FreeZone 6 Plus, Labconco, Kansas City, MO, USA) and stored at −80 °C until further sample preparation. Then, these were milled to a fine powder (MM301 Retsch, Haan, Germany; 30 Hz for 60 s), which was stored at −80 °C until extraction. Before and after lyophilization, moisture content was analyzed (MA51 Sartorius, Goettingen, Germany) to ensure that the remaining water content after lyophilization was below 5%.

#### 2.3.3. Reference Standard Mix

A 2 mg/L standard mix was prepared containing all standards listed in 2.1. Standards were dissolved and diluted with MeOH/ACN/water 1:1:2 (*v*/*v*/*v*) + 0.1% FA. For quantification, a 2000 µg/L stock was diluted to 1000 µg/L, 500 µg/L, 200 µg/L, 100 µg/L, 50 µg/L, 25 µg/L, 10 µg/L, 5 µg/L, and 2 µg/L.

### 2.4. Extraction and Lyophilization

#### 2.4.1. Extraction for Testing the Effect of Lyophilization on the Metabolome of Plant Material (Experiment PM)

To test the effect of lyophilization prior to sample extraction, fresh and lyophilized plant materials were extracted separately with the protocol of Doppler, et al. (2016) [9]. For extraction of lyophilized root samples, the extraction protocol used for fresh weight samples was adjusted (amount of extracted root material was reduced compared to the original protocol according to the lower water content) to obtain approximately equal abundances for the detected metabolites compared to fresh samples. Both the appropriate proportion of water as well as the sample to solvent ratio were tested and confirmed (data not shown). For fresh root samples, 100 mg of homogenized roots were mixed with 1 mL of pre-cooled MeOH/ACN 1 + 1 (*v*/*v*) mixed with water (3 + 1, *v*/*v*) + 0.1% formic acid. For lyophilized root samples, 10 mg of homogenized roots were mixed with 90 µL of pre-cooled ELGA water to achieve a water content similar to fresh roots (90%) as well as 1 mL of a pre-cooled mixture of MeOH/ACN 1 + 1 (*v*/*v*) mixed with water (3 + 1, *v*/*v*) + 0.1% formic acid.

Extracts were vortexed for 10 s and put into an ultrasonic bath (47 kHz, 105 W) for 15 min. Next, extracts were centrifuged (14,000 rpm, 4 °C) for 10 min. Supernatants were transferred into a fresh test tube and only then were the ^13^C extracts added to the native extracts (1 + 1, *v*/*v*) to perform internal standardization. Organic solvent content was reduced to 50% (*v*/*v*) by adding water + 0.1% formic acid. Extracts were then vortexed for 10 s and centrifuged again (14,000 rpm, 4 °C) for 10 min. The supernatants were transferred into HPLC vials for LC-HRMS measurement. In total, five replicates containing mixed extracts of native and ^13^C-labelled fresh roots, as well as five replicates containing mixed extracts of native and ^13^C-labelled dried roots, were prepared with this protocol for both extraction solvents, thereby comparing the metabolic composition of extracts of dried roots to that of fresh roots.

To compare different solvents, the reconstituted measurement samples were prepared independently, starting from the dried and fresh root materials with pure ELGA water or acidified MeOH/ACN, respectively.

#### 2.4.2. Extraction for Testing the Effect of Lyophilization When Used for Extract Concentration (Experiment EC)

To investigate the performance of extract concentration by lyophilization, both an untargeted approach employing native and ^13^C-labelled wheat roots and a targeted approach consisting of a mix of 15 reference standards were used.

For the untargeted approach, fresh and lyophilized plant materials were extracted separately with an adapted version of the extraction protocol of Doppler, et al. (2016) [9]. For fresh roots, 1 g of homogenized ^13^C-labelled and 1 g of native root samples were weighed into separate 15 mL Greiner tubes. Then, 10 mL of pre-cooled ELGA water was added for extraction. For lyophilized root samples, 150 mg of homogenized ^13^C-labelled and 150 mg of native lyophilized root samples were weighed into separate 50 mL Greiner tubes and 1350 µL of pre-cooled ELGA water were added to achieve a water content (approximately 90%) similar to fresh root samples. Then, 15 mL of pre-cooled ELGA water was added for extraction. The extracts were vortexed for 10 s and put into an ultrasonic bath (47 kHz, 105 W) for 15 min, followed by centrifugation (4000 rpm, 4 °C) for 20 min. Supernatants were transferred into a fresh test tube and only then were the extracts of either fresh or dried ^13^C-labelled roots added to extracts of the respective fresh or dried native roots (1 + 1, *v*/*v*) to perform internal standardization. These mixed extracts were then vortexed for 10 s and centrifuged again (4000 rpm, 4 °C) for 20 min. Supernatants were transferred into HPLC vials for LC-HRMS analysis. In total, 5 samples of aqueous extracts of fresh native and fresh ^13^C-labelled roots, as well as 5 samples of aqueous extracts of dried native and dried ^13^C-labelled roots were prepared with this protocol.

#### 2.4.3. Lyophilization of Extracts (Experiment EC)

Water extracts of fresh and lyophilized roots were then split into aliquots of 600 µL to perform experiment EC for fresh and lyophilized roots separately. Next, different dilution levels (1×, 2×, 4×, 8×, 16×, and 32×; each 5 replicates) were established with pure ELGA water to simulate samples with low metabolite concentrations (e.g., root exudates or supernatants of microbial cultures). Dilution of the reference standards was performed with a mix of MeOH/ACN/water 1:1:2 (*v*/*v*/*v*) + 0.1% FA. Prior to lyophilization, extracts were frozen at −80 °C and lyophilized at −80 °C. Then, the dilution series of standards was lyophilized to dryness and reconstituted in MeOH/ACN/water (1:1:2) + 0.1%FA to theoretically achieve the same concentration of the original samples (before dilution). In total, this protocol yielded 5 non-diluted samples, 5 diluted samples, and 5 concentrated samples for each dilution factor and for the root and standard samples. The dilution series of root extracts was lyophilized to dryness and reconstituted in water to theoretically achieve the same concentration of the original extracts (before dilution). All root samples (original, diluted, and lyophilized) were diluted with MeOH 1:1 (*v*:*v*) prior to measurement. Samples of the original extract, diluted extract, and lyophilized were used for LC-MS analysis. The metabolic composition of concentrated (lyophilized) extracts was compared to the original extracts. Therefore, the composition of the labelled material can also potentially be changed by the concentration step together with the native constituents, making the labelled material a relative reference, which compensates for metabolic changes by lyophilization.

The same steps were carried out for the standard mix and for a blank sample, consisting of pure solvent for each dilution factor.

### 2.5. LC-HRMS Analysis

The extracts were analyzed using an UHPLC system (Vanquish) coupled to an Orbitrap Q Exactive HF mass spectrometer (Thermo Scientific, Bremen, Germany) equipped with a heated electrospray ionization source. For chromatographic separation, a C18 reversed-phase column (3.5 μm; 2.1mm × 150 mm; XBridge^®^; Waters; Milford, MA, USA) was used. Autosampler temperature was set to 10 °C and the column temperature was set to 25 °C. For each sample, 10 µL were injected. The sequence order of samples was randomized. Water containing 0.1% formic acid (eluent A) and methanol containing 0.1% formic acid (eluent B) were used as eluents. Two chromatographic methods (short and long) were used. Short gradient (for root samples): A linear gradient with a constant flow of 250 µL/min was used for elution starting with 10% eluent B and continuously increasing B to 100% at 10 min after an initial hold time of 1 min. After a hold time of 3 min, the column was re-equilibrated for 7 min at 10% eluent B. Long gradient (for standards): A linear gradient with a constant flow of 250 µL/min was used for elution starting with 10% eluent B and continuously increasing B to 100% at 30 min after an initial hold time of 2 min. After a hold time of 5 min, the column was re-equilibrated for 8 min at 10 % eluent B. Full scan MS measurements were acquired with fast polarity switching to generate positively and negatively charged ions with a scan range of *m*/*z* 100 to 1000 and a resolution of 120,000 FWHM. The auxiliary and sheet gas flow rates were set to 5 and 55 units. Spray voltage was set to 3500 V for positive and 3000 V for negative mode. For MS/MS measurements, sample-specific inclusion lists were generated for the positive mode and negative mode for all samples of experiment PM and for the original samples of experiment EC. For this, the data were first evaluated with MetExtract II and each detected plant feature was put on an inclusion list with a 0.5 min time window. All MS/MS measurements were performed in a positive and negative mode separately with a resolution of 60,000 FWHM. Collision energies were set to 20, 45, and 70 eV stepped.

### 2.6. Data Processing, Statistical Analysis, and Metabolite Annotation

LC-HRMS raw data files were converted to the mzXML format using MSConvert of ProteoWizard (v3.0.21292) and subsequently processed with MetExtract II [36] (parameter settings are provided in Supporting Information S1). MS/MS spectra were extracted for each experiment separately using the XCMS package (parameters are provided in Supporting Information S2). To combine several MSMS spectra of the same feature, the MSnbase package was used with the average method and a deviation of 20 ppm was allowed to match fragment spectra. MSMS spectra were exported to the MGF format. Compound classes were predicted with CANOPUS [39] (SIRIUS, v5.5.7) and molecular networking was performed using MetGem (v1.3.6). Default parameters were used. For reference standard quantification, XCalibur Quan Browser (v4.2) was used. For level 1 annotation (identified) metabolites of samples were considered as matched to standards when retention time shift was smaller than 10 s and principal ions shifted by less than 5 ppm *m*/*z* deviation. Statistical analysis was carried out in R (https://r-project.org, v3.5.3), which used only the relative abundances of the monoisotopic isotopologs of the native metabolite forms (from the native roots). Further information about the used parameters can be found in Supporting Information S3.

## 3. Results

Studies that aim to investigate the performance of technical aspects of untargeted metabolomics workflows, such as sample preparation, need to cover a broad, representative range of metabolites and compound classes, hence wheat plants were used as they are rich in secondary metabolites of different substance classes [40]. Thus, it was first checked if the employed root samples fulfilled this criterion.

A total of 511 truly wheat-derived metabolites (from 2424 ions) were detected across all samples. The inspection of datafiles originating from samples with no ^13^C material revealed a few false positives (less than 20), which were removed from the data. This was achieved using the previously developed isotope-assisted metabolomics workflow. The interested reader is referred to [37] for further details on the protocols. Subsequent metabolite annotation using SIRIUS and CANOPUS annotated the detected metabolites as different chemical classes, including fatty acids and conjugates thereof, phenolic acids, phenylpropanoids, coumarins, and monoterpenoids (Figure S1). Additionally, over 100 of the detected wheat metabolites were annotated based on LC-HRMS full scan measurements by comparing their chemical formulas (*m*/*z* values, adducts and number of carbon atoms; level 3, [41]). For example, BOA, HMBOA, HBOA, DIBOA-Glc, and chlorogenic acid were annotated in samples of both experiments.

With these representative samples for untargeted plant metabolomics experiments in hand, the next step was to investigate the effect of lyophilization during sample preparation. More specifically, the effect of lyophilization upon the metabolic composition of the samples was assessed by testing how many metabolites can no longer be detected in samples with a lyophilization step (lost metabolites) and how many and which metabolites have altered (decreased or increased) abundances.

### 3.1. Experiment for Testing the Effect of Lyophilization on the Metabolic Composition of Plant Material (Experiment PM)

#### 3.1.1. Comparison of Extraction Solvents of Fresh and Dried (Lyophilized) Roots

**Figure 2:**
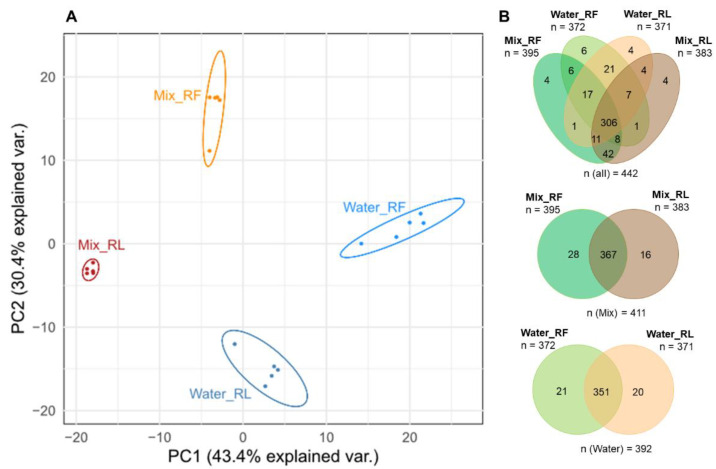
(**A**): PCA scores plot of extracts of fresh (RF) and dried (lyophilized; RL) roots extracted with water (Water) or acidified water/ACN/MeOH (Mix), respectively; (**B**): Venn diagrams showing the number of metabolites detected in the differently extracted root samples.

The impact of lyophilization upon metabolite abundances in different aqueous solutions was investigated. A total of 442 metabolites (from 2204 ions) were detected in the respective samples obtained from either fresh or dried root material, and for 289 of these metabolites, it was possible to acquire MS/MS spectra. Comparing the metabolic composition of fresh roots (Mix_RF) and dried (lyophilized) roots (Mix_RL) extracted with solvent mix and fresh roots (Water_RF) and dried roots (Water_RL) extracted with water, respectively, a principle component analysis (PCA) was carried out. The PCA scores plot (Figure 2A) shows a distinct separation of these four groups. The greatest difference along principle component 1 (PC1) was observed between Mix_RL and Water_RF. This can be expected since both extraction solvents as well as the water content differ between the investigated samples. The separation of Mix_RF and Water_RF, as well as Mix_RL and Water_RL, was also expected as already shown by Doppler, et al. (2016) [9]. Moreover, lyophilized (RL) and fresh (RF) root materials also separated after extraction with the same solvent (i.e., water or mix, respectively). Thus, it can be concluded that the influence of whether fresh or dried plant material is used has a comparably strong effect on the metabolic composition as the choice of extraction solvent.

With respect to the number of detectable metabolites (Figure 2B), the highest number of metabolites (395) was found in fresh roots extracted with solvent mix (acidified MeOH/ACN/water), while the lowest number of metabolites (371) was found in dried roots extracted with water. Overall, 306 metabolites (69%) were commonly detected. Only 7% of metabolites were found only in roots extracted with water and 11% in roots extracted with the solvent mix. Thus, it can be concluded that extraction with the solvent mix leads to only a marginally higher number of detectable metabolites.

As the effect of different solvents on the metabolic composition was investigated in more detail elsewhere (e.g., Doppler, et al. (2016) [9]), in the following, a more in-depth comparison of the metabolic composition between fresh and dried materials is presented.

#### 3.1.2. Comparison of Extracts of Fresh and Dried Roots

**Figure 3:**
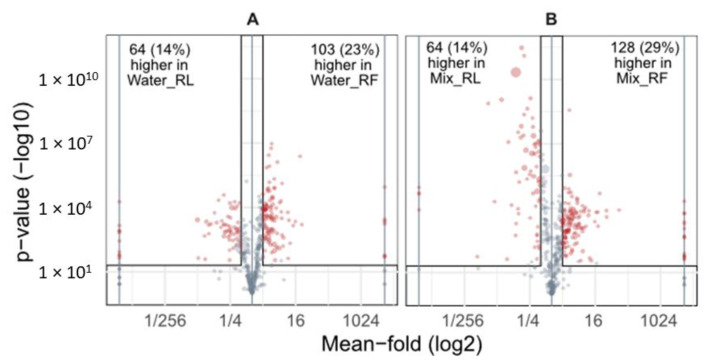
Volcano plots comparing samples obtained from dried and fresh root material extracted with either water or solvent mix. Grey dots indicate not significantly differing metabolite abundances, and red dots and numbers indicate significantly differing metabolite abundances (*p*-value < 0.05, mean-fold at least 2 or less than 0.5). (**A**) compares fresh and dried roots after extraction with water. (**B**) compares fresh and dried roots extracted with solvent mix (acidified MeOH/ACN/water).

The effect of lyophilization prior to sample extraction was investigated by comparing the extracts of the lyophilized samples to those of the samples from the fresh roots. This was conducted for both solvents. If lyophilization had no effect on sample composition and sample reconstitution worked equally well as extraction of metabolites from fresh roots, all fold changes in the volcano plots should be approximately 1 (i.e., no difference in metabolite abundance). A median fold change of 1.21 for roots extracted with solvent mix and 1.25 for water extracts indicates that on average, there is a slight tendency towards higher metabolite abundances in extracts of fresh roots (red dots in Figure 3). This suggests that lyophilization affected the metabolic composition of fresh root samples and that lyophilization was not equally effective for all of the metabolites present. As much as 37% (in the samples extracted with water) and 43% (in the samples extracted with the solvent mixture) of the metabolites detected had significantly lower or higher abundances in the extracts of the dried roots compared to extracts of the fresh roots. Here, the highest observed differences were as much as a 16-fold increase or decrease in the samples obtained from dried material in comparison to fresh material. Additionally, Figure 2B showed that 28 metabolites (7%) were detected only in fresh roots extracted with solvent mix, e.g., farnesyl acetat (annotated level 3) and 16 metabolites (4%) only in dried roots extracted with solvent mix, e.g., p-hydroxybenzaldehyde and D-pinitol (annotated level 3). For example, DIBOA, DIBOA-Glc, DIMBOA-Glc, indole-3-acetic acid, and isoleucine (annotation level 3), as well as tryptophan and ferulic acid (identified) showed significantly higher abundance in fresh roots compared to the corresponding extracts of dried roots. In contrast, phenylalanine (identified) as well as HBOA (annotation level 3) did not show significantly differing abundances between fresh and dried roots. Furthermore, as the number of affected metabolites and the extent to which their abundances were changed were comparable between the two tested extraction liquids, the average performance of lyophilization appeared to be independent of the used solvent.

The metabolic composition of the tested roots is largely unknown; nevertheless, many different compounds were putatively annotated with CANOPUS (e.g., polyphenols, fatty acids). To investigate whether lyophilization discriminates certain compound classes, the distribution of the assigned compound superclasses and pathways were compared between fresh and dried roots (Figure S2). For this, metabolites of a different superclass and pathway assigned with CANOPUS were split into groups according to their classification in the volcano plot (i.e., not significantly altered, and significantly higher in fresh or lyophilized samples, respectively; Figure 3). Similar distributions of compound classes between the experimental groups were observed, which is not indicative of a particular compound class to be systematically affected by the lyophilization procedure. A few superclasses were only detected in a single experimental group (e.g., peptide alkaloids in Mix_RF), but due to the low number of metabolites assigned as peptide alkaloid, this classification does not allow for concluding a generalization for the particular groups. Some superclasses with higher numbers of metabolites partly show a slightly lower frequency, e.g., phenolic acids, fatty acids and conjugates, phenylpropanoids, small peptides and monoterpenoids, or a higher frequency, e.g., fatty acyl glycosides and saccharides, of metabolites after lyophilization.

**Figure 4:**
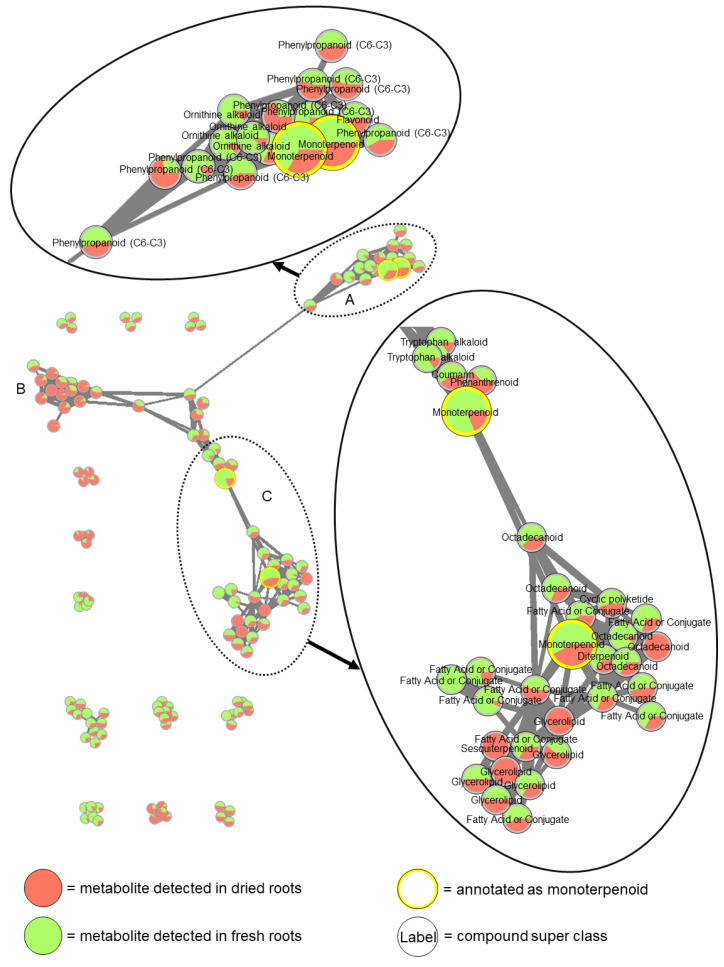
Molecular networks of metabolites detected (positive mode) in fresh roots (Mix_RF) and dried roots (Mix_RL) extracted with solvent mix. The pie charts illustrate the peak areas of the metabolite in the respective groups. Node names are according to superclass obtained from CANOPUS. All monoterpenoids are highlighted with a yellow node border and increased node size. Edge thickness is proportional to cosine score. Cutoff for cosine score was set to 0.7.

Molecular networking (Figure 4) was performed for fresh and dried roots extracted with solvent mix. Monoterpenoids, which are potential volatile compounds [42], are highlighted with a yellow node border and increased node size. A comparison of the peak areas of native features showed that the abundances of the monoterpenoids were in most cases reduced in dried samples (green dominates the corresponding circles) compared to fresh roots, indicating that their partial loss is caused by their volatility. A clustering of compounds, which were decreased (network A with dominating green areas, Figure 4) or increased (network B with dominating red areas, Figure 4) after lyophilization, is partly observed, suggesting the effect of lyophilization being structure-related to a certain extent.

In summary, while only few metabolites (5% in water extracts and 7% in solvent mix extracts) were completely lost in samples obtained from lyophilized material (Figure 2B), the fraction of metabolites having significantly altered concentration levels is much higher (167 metabolites (37%) in water extracts and 192 metabolites (43%) in solvent mix extracts; Figure 3). In our study, compound class analysis was not indicative for particular compound classes to be systematically biased by the lyophilization procedure. Still, molecular networking suggests that the partial loss of compounds by lyophilization is related to metabolite structure to a certain extent.

### 3.2. Effect of Lyophilization on the Metabolic Composition of Extracts after Concentration (Experiment EC)

In this experiment, it was investigated whether concentrating aqueous extracts to dryness with lyophilization and the subsequent reconstitution to the volume of the original extract prior to dilution can influence the metabolic composition and/or metabolite abundances in the resulting measurement solutions. To this end, extracts of fresh roots were diluted with water up to 32-fold to simulate liquid samples that contain metabolites at very low concentrations and then concentrated via lyophilization. Theoretically, if lyophilization worked without bias and equally well for all compounds, the measured concentrations should be identical to those obtained for the original extracts (i.e., prior to dilution). The extracts, which were diluted, lyophilized, and reconstituted, are referred to as “concentrated extracts” in this study.

In LC-HRMS-data of the fresh root extracts, 309 metabolites (from 1346 ions) were detected, and for 156 of those, LC-HRMS/MS spectra were acquired. In addition to extracts of fresh roots, reconstituted freeze-dried roots were also used in a parallel experiment to investigate if a second lyophilization step, the concentration of extracts, leads to a further alteration of both type and amount of metabolites. In LC-HRMS-data derived from the freeze-dried and reconstituted root extracts, a total of 342 metabolites (from 1786 ions) were detected, and for 167 of these, MS/MS spectra were acquired. Furthermore, a mix of authentic reference standards was also used with a similar experimental approach.

**Figure 5:**
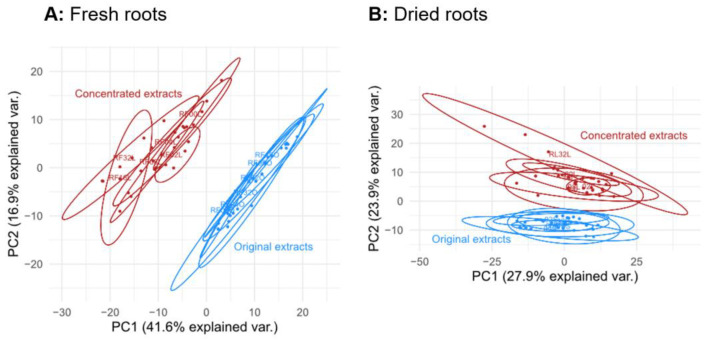
PCA scores plot of concentrated (diluted, lyophilized, and reconstituted) and original extracts of fresh (**A**) and dried (**B**) roots (replicates of each group are within the ellipses).

Original and concentrated, reconstituted extracts of fresh roots separated in a multivariate PCA scores plot along its first principal component (Figure 5A), indicating the difference of the metabolic profiles between original and concentrated extracts. In contrast, the PCA scores plot of original and concentrated extracts of previously freeze-dried root samples showed that concentrated and original extracts are less different and are separated not by PC1, but rather along PC2 (Figure 5B). This indicates that the initial stabilization of crude plant material by lyophilization potentially already changed the metabolic composition of the plant material and therefore partly masked the effect of concentrating and the reconstitution of the extracts of those roots. In summary, this indicates a reduced alteration of sample composition caused by lyophilization, which is expected since both the original and the concentrated extracts were obtained from already previously dried (lyophilized) material, and thus the biochemical composition was similarly affected by this technique in both types of samples. However, it seems the repeated lyophilization of the already previously freeze-dried roots altered the metabolic composition once again to a certain extent. The original and concentrated standard mix also separated slightly in a PCA (Figure S4), but groups still overlap. Furthermore, all diluted extracts show the expected pattern in the PCA for the standard samples, and the fresh and dried root samples (Figures S4, S6 and S9), with higher dilution factors separating more distinctly from the original and concentrated extracts in a quasi linear manner, confirming that the dilution and concentration step worked as expected.

**Figure 6:**
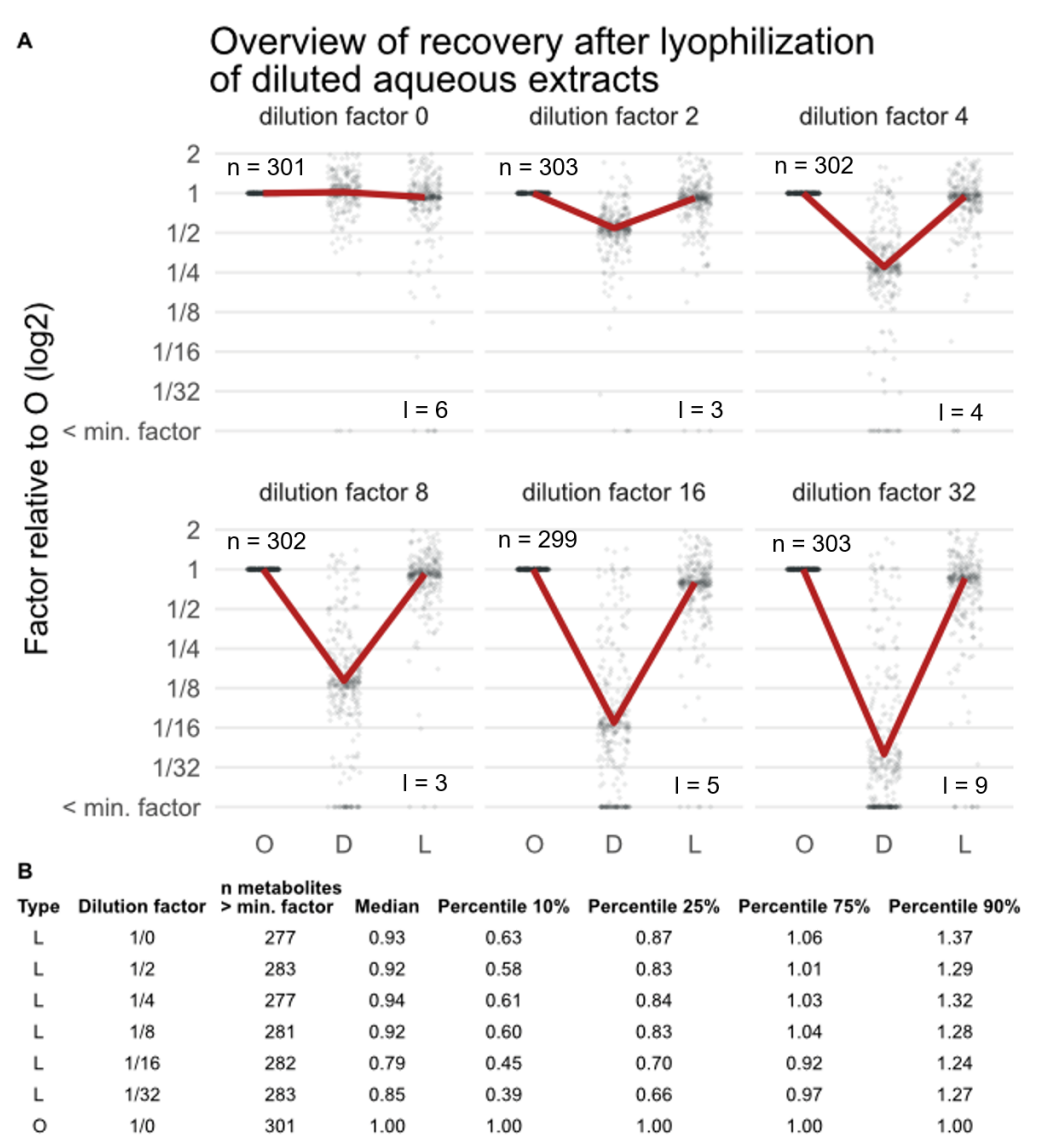
(**A**): Overview of metabolite recovery of extracts of fresh roots before lyophilization (O), after dilution (factors 0 to 32; D), and the recovery of this factor after concentrating by lyophilization and reconstitution (L); n indicates the number of metabolites detected in O, while l indicates the number of metabolites that are no longer detected in L. The red line shows the mean value of all metabolites, and each dot represents a metabolite. (**B**): Overview of how many metabolites were detected (> min. factor) in the respective sample, median, and 10-, 25-, 75-, and 90-percentiles.

The next step was to check the recovery rate after sample concentration and reconstitution. More than 95% of the metabolites present in the original extracts were also detected in the concentrated extracts of fresh roots, and the expected dilution factors are largely re-established after the lyophilization and reconstitution step for most metabolites (Figure 6). However, some metabolites clearly have different recoveries, especially at higher dilution/enrichment factors. The diluted samples showed the expected pattern for the comparison of diluted and original extracts of fresh roots (e.g., a factor of 1/4 relative to the original sample extract for factor 4 is reached). However, 3–9 metabolites (less than 5%) were not detected any longer in those samples (dots at ‘< min. factor’). Comparing peak areas of concentrated extracts relative to original extracts of fresh roots showed that for dilution factor 4, the sample composition is almost restored (i.e., a median relative factor of 0.94 is reached). With increasing dilution/enrichment factor and lyophilization time, reduced recovery rates were observed, with an average of 85% for extracts that were diluted by a factor of 32 prior to lyophilization. Similar results were observed for samples of dried roots (Figure S10) and the standard series (Figure S3). All standard compounds present in the original standard mix were also detected in the concentrated and reconstituted standard mix, but the obtained abundances were partly lower than theoretically expected. Especially for standard mixtures with higher dilution/enrichment factors, the recoveries were reduced to an average of 74% for dilution factor 32.

The tendency to reduce recovery rates with an increasing dilution factor can also be observed in a univariate comparison of the abundance levels for individual metabolites before and after lyophilization. This is illustrated with Volcano plots in Figure S7 for fresh roots extracted with solvent mix. The comparison of original (O) and concentrated (lyophilized, L) extracts showed slight but decreasing lower abundances in the concentrated and reconstituted extracts, indicating that, on average, the abundances of the metabolites were marginally decreased in concentrated extracts compared to the original extracts. Additionally, the number of significantly differing metabolite abundances increased slightly with the dilution factor, up to 14% (43 metabolites; 11 of which have a higher and 32 have a lower abundance after the lyophilization and reconstitution step) for dilution factor 32 (Figure S7).

**Figure 7:**
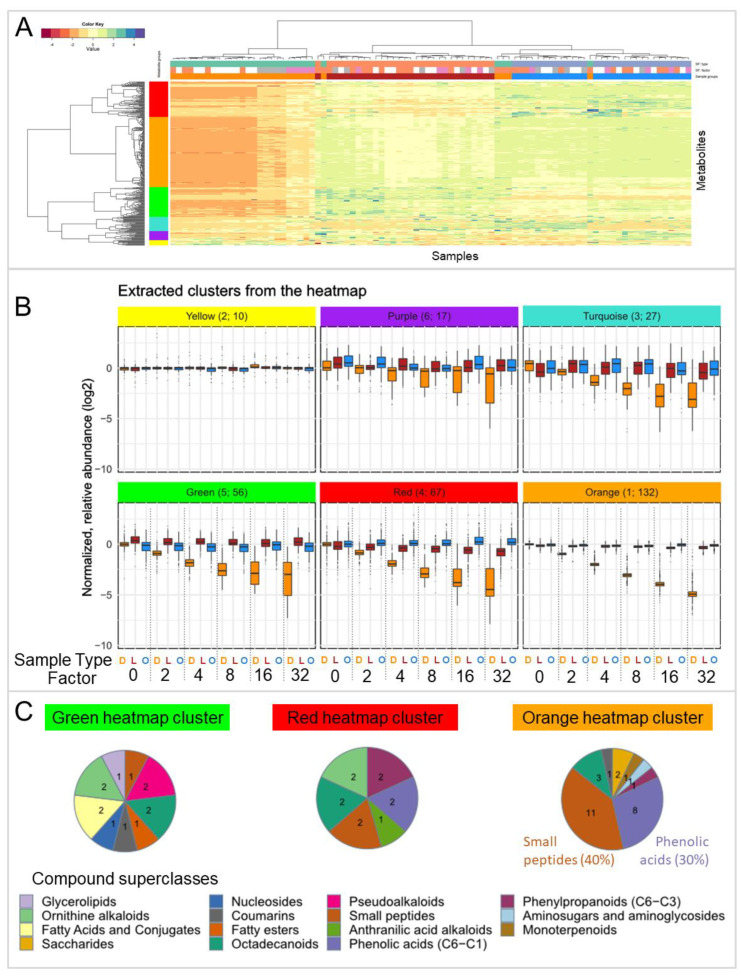
(**A**): Heatmap of original (O, blue), diluted (D, orange), and concentrated (diluted, lyophilized, and reconstituted; L, red) fresh root extracts. (**B**): The metabolite dendrogram was cut into six subclusters. Numbers in brackets are cluster number and number of compounds belonging to the respective cluster. Normalized abundances of original and concentrated extracts are shown relative to original samples. (**C**): Pie charts of counts of compound superclass (derived from CANOPUS) of selected extracted clusters from the heatmap. Compound superclass probability cutoff was set to 0.6.

In order to inspect the recovery of individual metabolites and whether their behavior is structure class-dependent, a heatmap based on fresh root extracts (Figure 7A) was generated. Subsequently, the metabolite dendrogram was cut in a supervised manner into six clusters. For each cluster, the abundances of the metabolites of diluted and concentrated (diluted, lyophilized, and reconstituted) extracts are illustrated relative to the mean of the original extracts as boxplots (Figure 7B). Five of the six clusters show the expected abundance patterns of decreasing peak areas with increasing dilution factor. However, metabolites of cluster 2 (yellow) at the bottom of the heatmap did not change with increasing dilution. Therefore, these compounds most likely represent metabolites that were false positively detected during automated data processing.

For cluster 1 (orange), the abundance of the compounds in concentrated extracts is almost identical to the original extracts, indicating that lyophilization increased the compounds’ respective abundance of the diluted extracts to the original level successfully and almost completely. The recovery rate of the compounds in cluster 1 for factor 32 is 0.86 (median). This cluster is the largest in the metabolite dendrogram, covering 132 metabolites, including tryptophan and phenylalanine (identified), as well as isoleucine and HBOA-glc (annotated level 3). Tryptophan and phenylalanine showed identical behavior in both the untargeted experiment (wheat roots) and the targeted experiment (reference standards).

The four other clusters (red, green, turquoise, and purple) showed similar behavior to the orange cluster, but higher deviation among the metabolite abundances in the different groups and also increased or decreased recovery rates in the concentrated samples. The recovery rate of the compounds underlying cluster 6 (purple) factor 32 is 1.02 (median), but the peak areas of diluted extracts in this cluster seem to be larger than expected on average, which is also observed (but less pronounced) in cluster 3 (turquoise). For cluster 3 and 4 (turquoise and red), the concentrated extracts did not fully reach the same value as the original extracts and therefore show reduced concentrations and recovery rates for factor 32 of only 0.46 and 0.47 (median). Cluster 4 (red) showed lower precision in comparison to cluster 1 (yellow) and includes linolenic acid and ferulic acid (identified), as well as HMBOA-glc (annotated level 3). For cluster 5 (green) the concentrated extracts reached higher values than the original extracts, meaning that these compounds were concentrated more than in theory. The diluted extracts underlying this cluster do not show the expected linear relationship and increasingly differ from the expected values with increasing dilution factor. The reason for this could be an analytical problem, a loss of compounds at low concentrations (e.g., through absorption onto test tube walls), or the chromatographic peaks being too small to be integrated properly. The recovery rate of the compounds underlying cluster 5 for factor 32 is 1.28 (median). Cluster 5 includes HBOA and HMBOA (annotated level 3).

This similar trend of metabolites in the same cluster raised again the question of whether the respective metabolites have correlated structural features. Therefore, pie charts (Figure 7C) were generated for heatmap cluster 1 (orange), cluster 4 (red), and cluster 5 (green) to compare compound superclasses (derived from CANOPUS with a minimum probability score of 0.6 [39]). Cluster 1 (orange) showed a high number of small peptides and phenolic acids. However, in general, the annotations were diverse, comprising of many different compound classes, which does not allow deriving certain compound classes to be systematically affected by the lyophilization and reconstitution procedure. Moreover, organizing the significant and not significant metabolites in a feature plot (Figure S8) shows also no systematic correlation between original and lyophilized samples, indicating no relation of the observed effect with the polarity of compounds.

Furthermore, 11 of the 15 compounds from the targeted experiment were not detected in the wheat root samples (experiment PM). For schaftoside, 5-deoxy-5(methylthio)adenosine, d-desthiobiotin, reserpine, deoxynivalenol, and scopoletin, the abundance of the compounds in lyophilized samples is (approximately) equal to original standards (Figure S5). For chlorogenic acid and 5-methoxy-3-indoleacetic acid, the concentration of compounds in concentrated extracts is slightly reduced after lyophilization at higher dilution factors. For ethyl ferulate, 3-(4-hydroxyphenyl)acrylate, 3-(4-hydroxyphenyl)propionate, and linoleic acid, the abundance of compounds in concentrated extracts is reduced in comparison to original standards.

## 4. Discussion

### 4.1. Discussion of General Results

Several hundred truly plant-derived metabolites from diverse compound classes (e.g., phenolic acids, monoterpenoids, fatty acids, and conjugates) in a wide range of polarity and abundances were detected with the help of the stable isotope-assisted workflow. All results are based on the occurrence of a metabolite in at least three out of five replicates to be included in the statistical evaluation. Consequently, the investigated root samples are well suited for testing the performance of lyophilization during sample preparation.

### 4.2. Experiment for Testing the Effect of Lyophilization on the Metabolic Composition of Plant Material (Experiment PM)

First, the effect of lyophilization on the metabolic composition of plant material prior to sample extraction was studied. An expected difference of aqueous extracts and solvent mix extracts obtained from either fresh or dried roots was observed (Figure 2A) and can be attributed to different extraction efficiencies with different solvents, which lead to the extraction of marginally higher numbers of detectable metabolites in root material extracted with solvent mix. These results are in agreement with previous studies, e.g., Doppler, et al. (2016) [9] and thus, the focus was directed to the comparison of extracts obtained from fresh and dried (lyophilized) roots, which are also separated distinctly (Figure 2A), indicating that the freeze-drying of the roots had a significant effect on their metabolic composition. Based on Figure 2B, slightly more metabolites were detected in extracts of fresh than in dried (lyophilized) roots, and 21–28 metabolites (5–7%) of metabolites detected in the extracts of the fresh roots were completely lost in the extracts of freeze-dried root samples, e.g., farnesyl acetate (annotated level 3). This can be considered problematic for a technique that is described as the “golden standard” for gentle drying and sample stabilization in the literature and has to be taken into account when performing untargeted metabolomics studies. In addition to that, 16 metabolites (4%) were detected only in solvent mix extracts of dried roots, e.g., p-hydroxybenzaldehyde and D-pinitol (annotated level 3), indicating that some metabolites were altered through the process of lyophilization (e.g., metabolite A, present in fresh samples, is transformed to metabolite B during lyophilization).

In addition to the complete loss of metabolites, the relative abundance of a much larger number of metabolites (37% for water extracts and 43% for solvent mix extracts) was significantly affected by lyophilization when compared to the extracts of fresh roots. Here, the highest observed differences were as much as a 16-fold increase or decrease in the samples obtained from dried material in comparison to fresh material. Oikawa and colleagues also observed that lyophilization caused changes in metabolite levels [33]. In our study, a trend towards higher abundances in extracts of fresh roots was observed for both extraction solvents. Solvent mix extracts showed 192 metabolites with significantly altered abundances, 64 of which showed higher abundance in extracts of dried roots and 128 showed higher abundance in extracts of fresh roots. This could be reasoned by different extraction efficiencies for the metabolites for either fresh or dried root material. Such strong differences can have a significant impact on the outcome of comparative metabolomics studies.

Comparing compound superclass distribution and pathways between solvent mix extracts of fresh and dried roots obtained through CANOPUS showed an overall similar distribution between the experimental groups, which is not indicative of a particular compound class being systematically affected by the lyophilization procedure. However, this is, to a certain extent, also not too surprising, since compound classes are broad classifications of chemical structures that might not correlate with the physical properties or specific structural moieties that can explain deviating relative abundances of metabolites after lyophilization (for example, carboxylic acids comprise both fatty acids and phenolic acids). On the other hand, clusters of structurally similar metabolites formed in molecular networking analysis (Figure 4), indicating that the effect of lyophilization might indeed be structure-related.

Moreover, the assignment to compound superclasses showed many diverse classes with a lower frequency (e.g., phenolic acids, fatty acids and conjugates, phenylpropanoids, and small peptides and monoterpenoids) or a higher frequency (e.g., fatty acyl glycosides and saccharides) in samples from dried roots (Figure S2). Some compounds were annotated as monoterpenoids. Their abundances were partly reduced after lyophilization, as can be expected from the volatile character [42]. A partial reduction in abundance of phenolic acids was also observed in extracts of dried roots, which is consistent with the findings of García-Salas and colleagues, who reported that low temperature drying lowered the phenolic content in the example of lemons [26]. Other studies reported that the reduction in phenolic content was dependent on plant species, with total phenolics showing similar levels in lyophilized samples compared to frozen tissues in the cases of marionberry and corn, but lower levels in strawberry [43]. In our study, small peptides as well as fatty acids and conjugates also showed a partial reduction in abundance after lyophilization (Figure S2). Moreover, tryptophan (identified) and isoleucine (level 3) showed a significantly reduced abundance, while the amount of phenylalanine (identified) was not affected in extracts of dried root. Dunstan and colleagues reported similar levels of polar lipids (which included mostly phospholipids and glycolipids [44]), sterols, hydrocarbons, and free fatty acids in dried samples compared to control samples, but reported a reduction of 16:0, unsaturated octadecanoic acids, and triacylglycerols, as lyophilization can increase the difficulty with which lipids can be extracted from the freeze-dried samples with solvents [45]. Additionally, DIBOA, DIBOA-Glc, DIMBOA-Glc, and indole-3-acetic acid (level 3) showed significantly reduced abundances in extracts of dried roots, while HBOA did not show significantly different abundances. Possible explanations for reduced abundances of metabolites in extracts from lyophilized samples can be different extraction efficiencies from fresh and lyophilized plant material or partial loss of compounds by chemical reactions, such as oxidation in solution. Oikawa and colleagues observed a decrease in levels of organic acids after lyophilization, which they reasoned to be due to a pH shift because of lyophilization damaging cellular organelles [33].

In summary, the presented results indicate that using lyophilization to dry plant material for stabilization globally (i.e., broad spectrum of substance classes) affects a large number of all metabolites (up to 43%) significantly towards both lower but also higher abundances, and also leads to the loss of up to 7% of metabolites. Thus, it is important to know the extent of the alteration in metabolite abundances for subsequent interpretation in targeted (if compounds are investigated) as well as untargeted approaches. Since lyophilization of fresh plant material can affect the metabolic composition and/or solubilization of compounds during reconstitution of analytical samples, it is advisable to work either exclusively with dried or fresh plant samples. Otherwise the described bias is always present when comparing dried (lyophilized) samples to fresh samples. Ideally, the effects are tested for other matrices than the here investigated root materials as well.

### 4.3. Effect of Lyophilization on the Metabolic Composition of Extracts after Concentration (Experiment EC)

Next, it was investigated if concentrating aqueous extracts with lyophilization influences metabolic composition in the concentrated (diluted, lyophilized, and reconstituted) extracts.

In general, the results of extracts of pre-dried roots are very similar to those obtained for fresh roots, showing high reproducibility over all experiments. The concentrated extracts and the original extracts of fresh roots separated in a multivariate PCA scores plot (Figure 5), indicating that lyophilization of extracts affects the metabolite abundance levels compared to the fresh, undiluted extracts. On the other hand, when the original sample material was obtained from already dried plant roots, the original and the concentrated samples did not separate in the PCA scores plot that strongly. This indicates that metabolic composition was less affected during the second lyophilization step (i.e., concentration of the diluted extracts) and thus less variation between these and their original samples was present in the dataset.

Theoretically, if lyophilization worked without bias and equally well for all compounds, the measured concentrations should be identical to those obtained for the original extracts (i.e., prior to dilution). Indeed, high recovery factors were observed in the simulation study, which declined with an increase in the concentration factors, reducing median recovery rate across all metabolites to 0.75 for standard samples and to 0.85 for fresh root samples at an enrichment factor of 32. Moreover, the number of metabolites significantly deviating from the theoretical concentration factor increased with higher enrichment factors (up to 14% of all detected metabolites, Figure S7) for extracts of fresh roots. A potential explanation for this could again be altered/decreased solubility of compounds after lyophilization to dryness as well as chemical alterations during lyophilization and loss of volatile metabolites. Still, more than 95% of the metabolites present in the original extracts are also detected in the concentrated extracts of fresh roots and the expected dilution factors are almost re-established after the lyophilization and reconstitution step for the majority of the metabolites (Figure 6). For the set of authentic reference standards investigated in this study, generally, fatty acids, such as linoleic and linolenic acid, showed reduced abundance in concentrated and reconstituted samples compared to original standard solutions of the targeted approach, which is consistent with the findings of Oikawa, et al. (2011) [33] and Dunstan (1993) [44]. In the untargeted approach, the same behavior was observed for linolenic acid (identified). This might be explained by autoxidation or decreased re-solubilization efficiency. Additionally, the plant-derived nitrification inhibitors, methyl coumarate (methyl 3-(4-hydroxyphenyl)acrylate; an esterified derivative of p-coumaric acid), 3-(4-hydroxyphenyl)propionate, and methyl ferulate, showed reduced abundance after lyophilization and reconstitution. Chlorogenic acid showed reduced concentrations in concentrated samples with an increasing dilution factor. Interestingly, other studies reported an increase in chlorogenic acid after lyophilization of grapes, which they reasoned as a result of affected re-solubilization efficiency [46,47]. A similar, but less pronounced effect of lyophilization was also observed for 5-methoxy-3-indoleacetic acid, with metabolite recovery decreasing with higher dilution factors. A reduction in abundance in concentrated extracts is consistent with the finding of Oikawa, et al. (2011) [33], who investigated indoleacetic acid (plant hormone) in *Arabidopsis* plants and pear fruits. Amino acids, e.g., phenylalanine and tryptophan, have an equal abundance in lyophilized samples and original extracts in both the targeted and untargeted approach (level 1 annotation), indicating that lyophilization did not affect these compounds. Additionally, reserpine, an indole alkaloid, with tryptophan as the starting material in the biosynthetic pathway, showed similar results. In contrast, the abundance of schaftoside (flavonoid C-glycoside), scopoletin (hydroxycoumarin), desthiobiotin (imidazolidinone), 5-deoxy-5-methylthioadenosine (intermediate in the generation of adenine and methionine), and deoxynivalenol (trichothecene mycotoxin), were not affected by concentrating with lyophilization. No clear correlation between reduced or increased recovery rates and the metabolites’ polarity of chemical class annotation was observed, though.

In summary, the study showed that lyophilization achieves the desired concentration for most metabolites in a linear fashion, which is necessary for comparative metabolomics studies. Nevertheless, not all metabolites are perfectly concentrated, and up to 14% exhibit significantly increased or decreased median recovery rates with an increasing concentration factor. No strong indication was found that this effect of lyophilization is compound superclass specific or can be reasoned in a polarity of compounds. Therefore, it is recommended to test lyophilization for the compounds of interest.

## 5. Conclusions

Sample integrity, or at least control of biochemical alterations, is key for any metabolomics analysis for meaningful metabolite annotation and interpretation of results. Here, we evaluated lyophilization, a sample preparation step commonly used in many metabolomics studies. Surprisingly, freeze-drying, which is generally considered as a very gentle sample concentration and stabilization method, significantly altered the abundance of up to 43% of all metabolites of a broad spectrum of substance classes (e.g., monoterpenoids, phenolic acids, fatty acids, and small peptides) and also led to the complete loss of up to 7% of metabolites. Furthermore, the results indicate that structurally similar metabolites are affected similarly (molecular networks). The observed significant changes in the biochemical composition with both strongly increased and decreased abundances of the tested plant samples is also suggested to work either exclusively with dried or fresh plant samples. This also implies that quantification of such metabolites in samples obtained from lyophilized material might over or underestimate actual concentrations in fresh material.

With respect to the concentration of sample extracts by lyophilization, more than 95% of the detected metabolites were concentrated in a linear fashion, which is necessary for comparative metabolomics studies. However, still up to 14% of the detected metabolites exhibited significantly increased or decreased median recovery rates with increasing concentration factors, which can make their absolute quantification within targeted studies challenging. The affected metabolites did not belong to specific compound superclasses.

In summary, lyophilization is a powerful technique for untargeted metabolomics that allows for easy sample concentration and stabilization, but care must be exercised, as it can significantly change a large portion of the metabolome with respect to both chemical structures as well as relative abundances of biochemical constituents. This can be especially troublesome for untargeted approaches and needs to be considered for absolute quantification and also for comparison with other experiments, e.g., from literature or public repositories.

## Data Availability

The data presented in this study are openly available in GNPS/MassIVE at [https://doi.org/10.25345/C5TX35H06], reference number MSV000091769.

## Supplementary Materials

The following supporting information can be downloaded at: https://www.mdpi.com/article/10.3390/metabo13060686/s1. Figure S1: Distribution of metabolite superclass annotations according to the Natural Product Classifier obtained via SIRIUS. Figure S2: Distribution of obtained metabolite superclasses and pathway distribution annotations of fresh and dried roots extracted with extraction solvent mix. The frequency of metabolites annotated with the respective class are shown. Figure S3: A: Overview of recovery of standards after lyophilization (L). The recovery is given relative to the mean values of the sample before lyophilization (O, original sample). B: Overview of how many compounds were detected (>min. factor) in the respective standard mix, as well as the recoveries in median and percentiles of 10%, 25%, 75%, and 90%. Figure S4: PCA scores plot of diluted (D, orange), concentrated (L, red) and original (O, blue) standard samples. The ellipses indicate the 95% confidence intervals for each group calculated from the respective replicates. Left: all samples and groups; Right: only the original and concentrated samples. Figure S5: Box plots of the normalized relative abundances of each samples type (diluted (D), lyophilized (L) and original (O)) for each factor (0 to 32) of all standards. Figure S6: PCA scores plot of diluted (D, orange), concentrated (L, red) and original (O, blue) samples obtained from fresh roots. The ellipses indicate the 95% confidence intervals for each group calculated from the respective replicates. Left: all samples and groups; Right: only the original and lyophilized samples. Figure S7: Overview of univariate tests of extracts of fresh roots (RF) before lyophilization (O), after dilution (D), and the after lyophilization plus reconstitution (L) at different dilution factors (number in group name). Grey dots indicate non-significantly differing metabolite abundances, red dots indicate significantly differing metabolite abundances (p-value < 0.05, mean-fold at least 2 or less than 0.5). The plots in the first column compare the diluted to the original extracts for each of the tested dilution factors. The plots in the second column compare the diluted to the concentrated samples of the respective dilution factor. The plots in the third column compare the concentrated extracts of the respective dilution factor to the original extracts of dilution factor 0. Figure S8: Feature plot (retention time versus *m/z* value) based on the results of the volcano plot (Figure S7) of the comparison of original and concentrated (lyophilized) extracts of fresh roots of factor 32. Dot size is proportional to peak area. Figure S9: PCA scores plot of diluted (D, orange), lyophilized (L, red) and original (O, blue) samples obtained from lyophilized roots. The ellipses indicate the 95% confidence intervals for each group calculated from the respective replicates. Left: all samples and groups; Right: only the original and lyophilized samples. Figure S10: A: Overview of metabolite recovery of extracts of dried roots before lyophilization (O), after dilution (factors 0 to 32) (D), and the recovery of this factor after concentrating by lyophilization (L). n indicates the number of metabolites detected in O, while l indicates the number of metabolites that have no longer been detected in L. The red line shows the mean value of all metabolites and the dots represent metabolites. B: Overview of how many metabolites were detected (> min. factor) in the respective standard mix, median and 10-, 25-, 75-, and 90-percentiles. Supporting Information S1: Parameters for MetExtract II. Supporting Information S2: Parameters for XCMS processing. Supporting Information S3: Statistical analysis procedures.
