# Supplementary material for: Untargeted Plant Metabolomics: Evaluation of Lyophilization as a Sample Preparation Technique
Source: Metabolites. 2023 May 25;13(6):686. doi: 10.3390/metabo13060686 (PMC10300689; doi:10.3390/metabo13060686)
Supplement: Supplementary file 1 [file metabolites-13-00686-s001.zip › metabolites-2386632-supplementary.pdf]

Article

Untargeted Plant Metabolomics: Evaluation of Lyophilization as Sample Preparation Technique

Christina Maisl, Maria Doppler, Bernhard Seidl, Christoph Bueschl and Rainer Schuhmacher

Supplementary figures and information

Table of Contents

Figure S1.. 2
Figure S2.. 3
Figure S3.. 4
Figure S4.. 5
Figure S5.. 6
Figure S6.. 7
Figure S7.. 8
Figure S8.. 9
Figure S9.. 10
Figure S10.. 10
Supporting Information S1.. 12
Supporting Information S2.. 12
Supporting Information S3.. 12

Overview

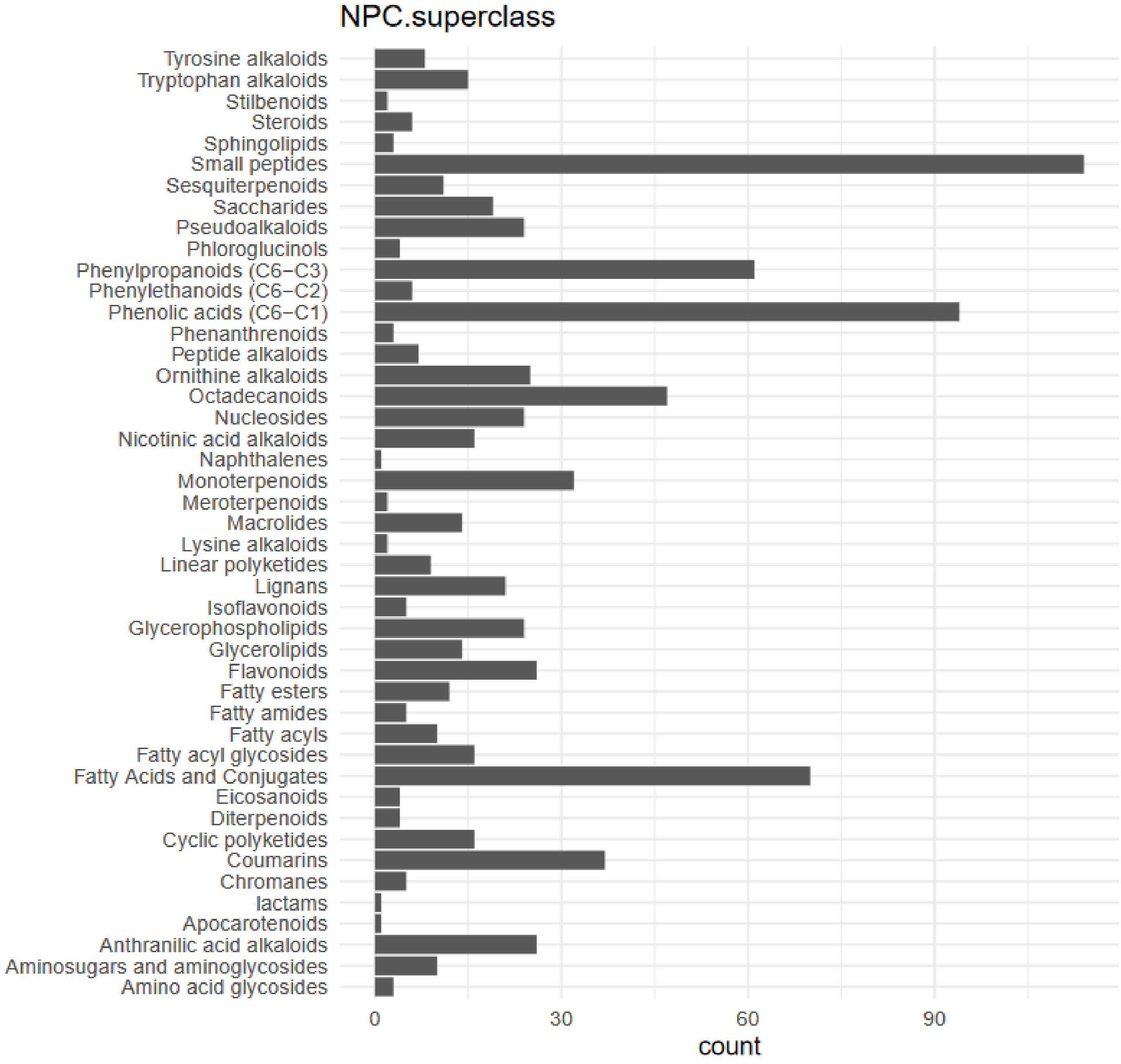

Figure S1. Distribution of metabolite superclass annotations according to the Natural Product Classifier obtained via SIRIUS.

Experiment PM

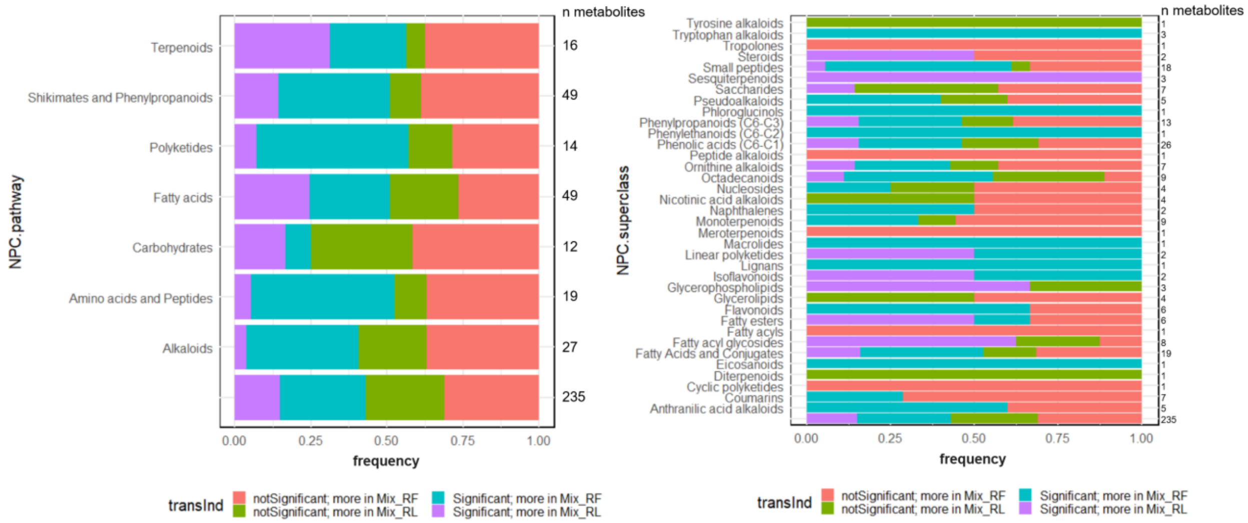

Figure S2. Distribution of obtained metabolite superclasses and pathway distribution annotations of fresh and dried roots extracted with extraction solvent mix. The frequency of metabolites annotated with the respective class are shown.

Experiment EC: Standard mix

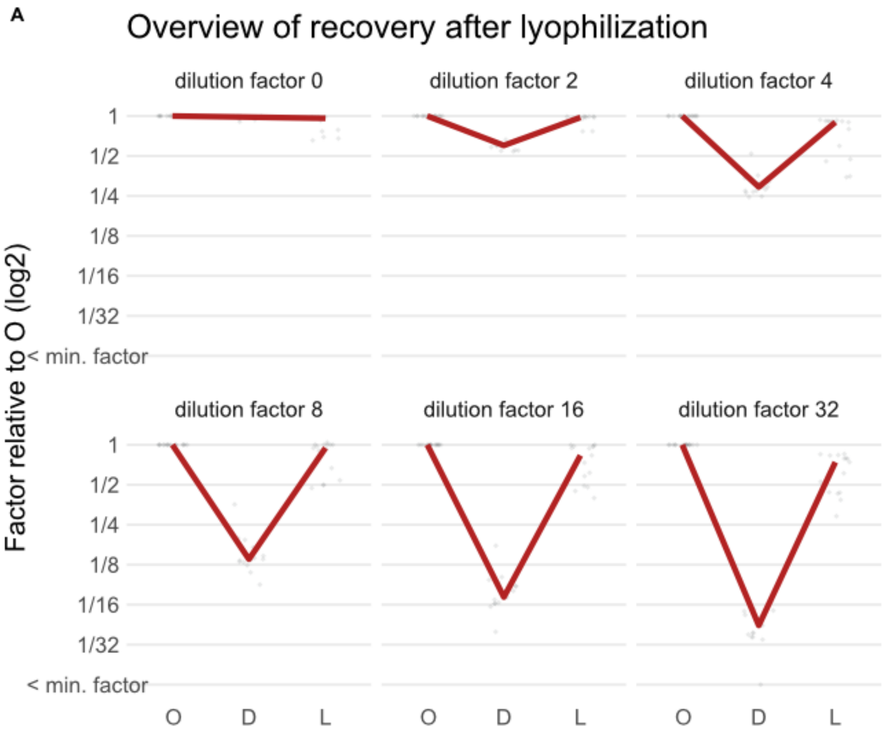

B

Type	Dilution factor	> min. factor	Median	Percentile 10%	Percentile 25%	Percentile 75%	Percentile 90%
L	1/0	15	0.96	0.68	0.78	0.97	0.98
L	1/2	15	0.98	0.81	0.95	0.99	0.99
L	1/4	15	0.90	0.39	0.53	0.92	0.93
L	1/8	15	0.95	0.50	0.60	1.00	1.00
L	1/16	15	0.83	0.46	0.54	0.97	0.99
L	1/32	15	0.74	0.40	0.44	0.81	0.84
O	1/0	15	1.00	1.00	1.00	1.00	1.00

Figure S3. A: Overview of recovery of standards after lyophilization (L). The recovery is given relative to the mean values of the sample before lyophilization (O, original sample). B: Overview of how many compounds were detected (> min. factor) in the respective standard mix, as well as the recoveries in median and percentiles of 10%, 25%, 75%, and 90%.

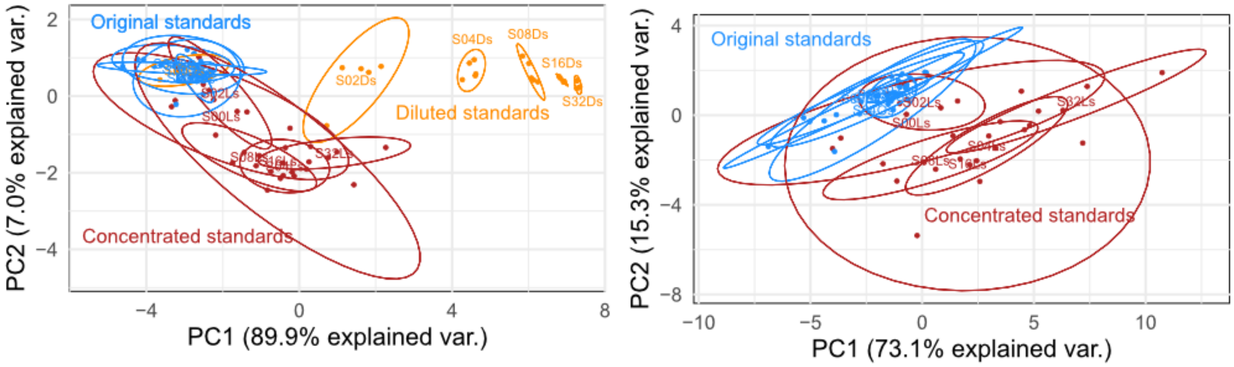

Figure S4. PCA scores plot of diluted (D, orange), concentrated (L, red) and original (O, blue) standard samples. The ellipses indicate the 95% confidence intervals for each group calculated from the respective replicates. Left: all samples and groups; Right: only the original and concentrated samples.

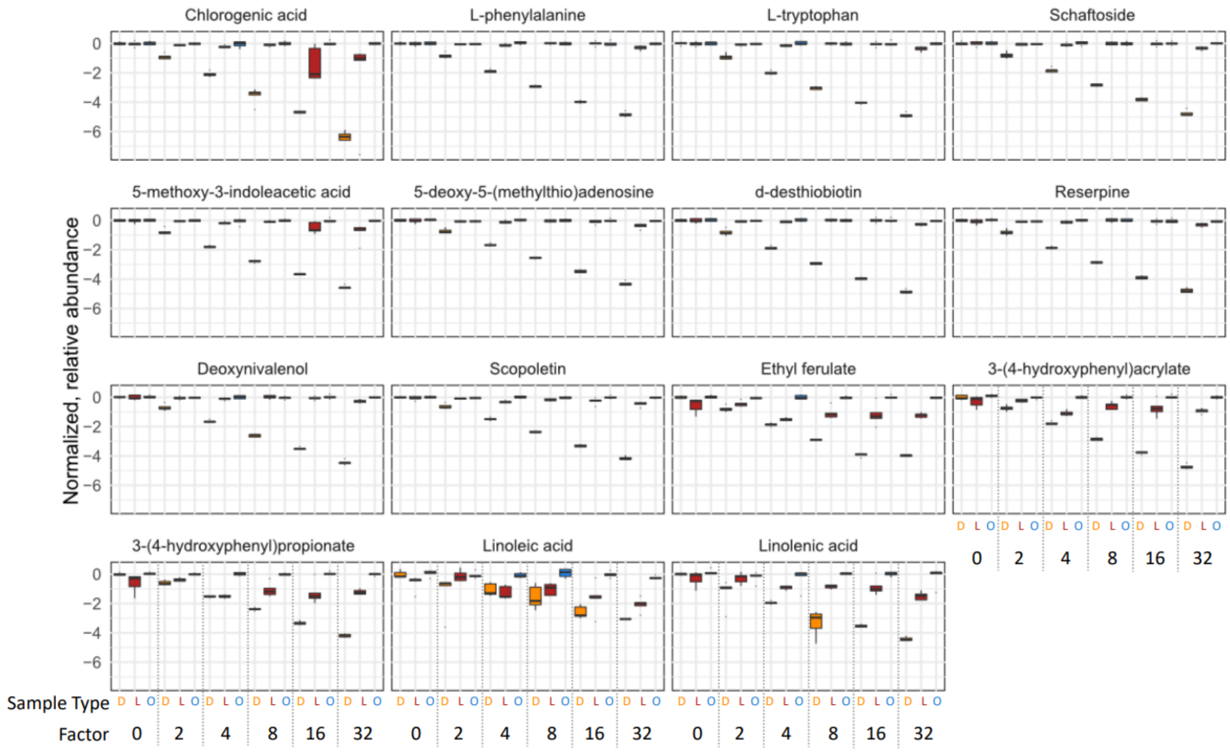

Figure S5. Box plots of the normalized relative abundances of each samples type (diluted (D), lyophilized (L) and original (O)) for each factor (0 to 32) of all standards.

Experiment EC: Fresh roots

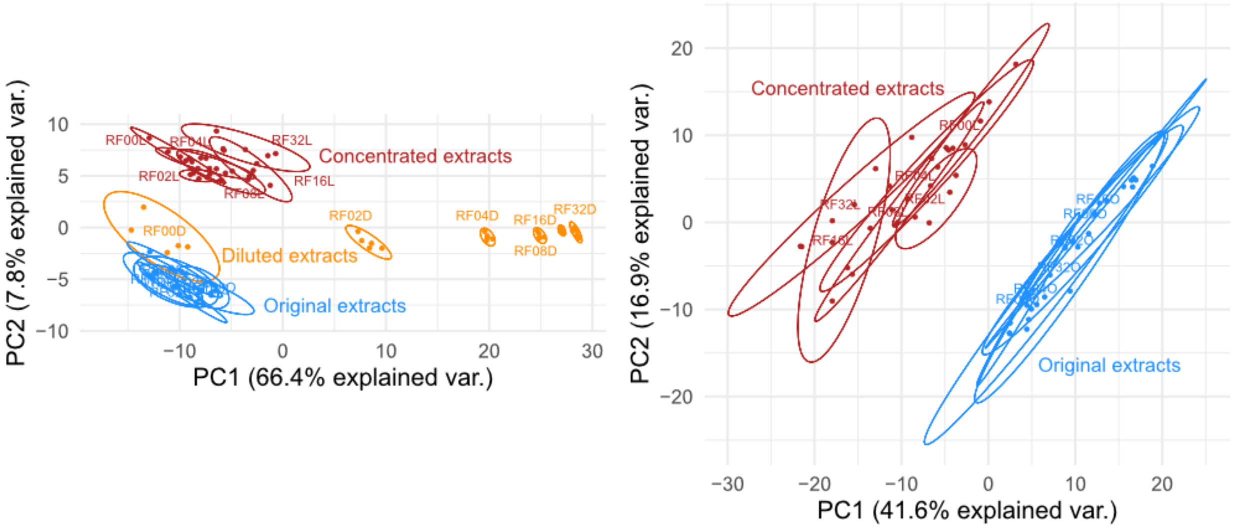

Figure S6. PCA scores plot of diluted (D, orange), concentrated (L, red) and original (O, blue) samples obtained from fresh roots. The ellipses indicate the 95% confidence intervals for each group calculated from the respective replicates. Left: all samples and groups; Right: only the original and lyophilized samples.

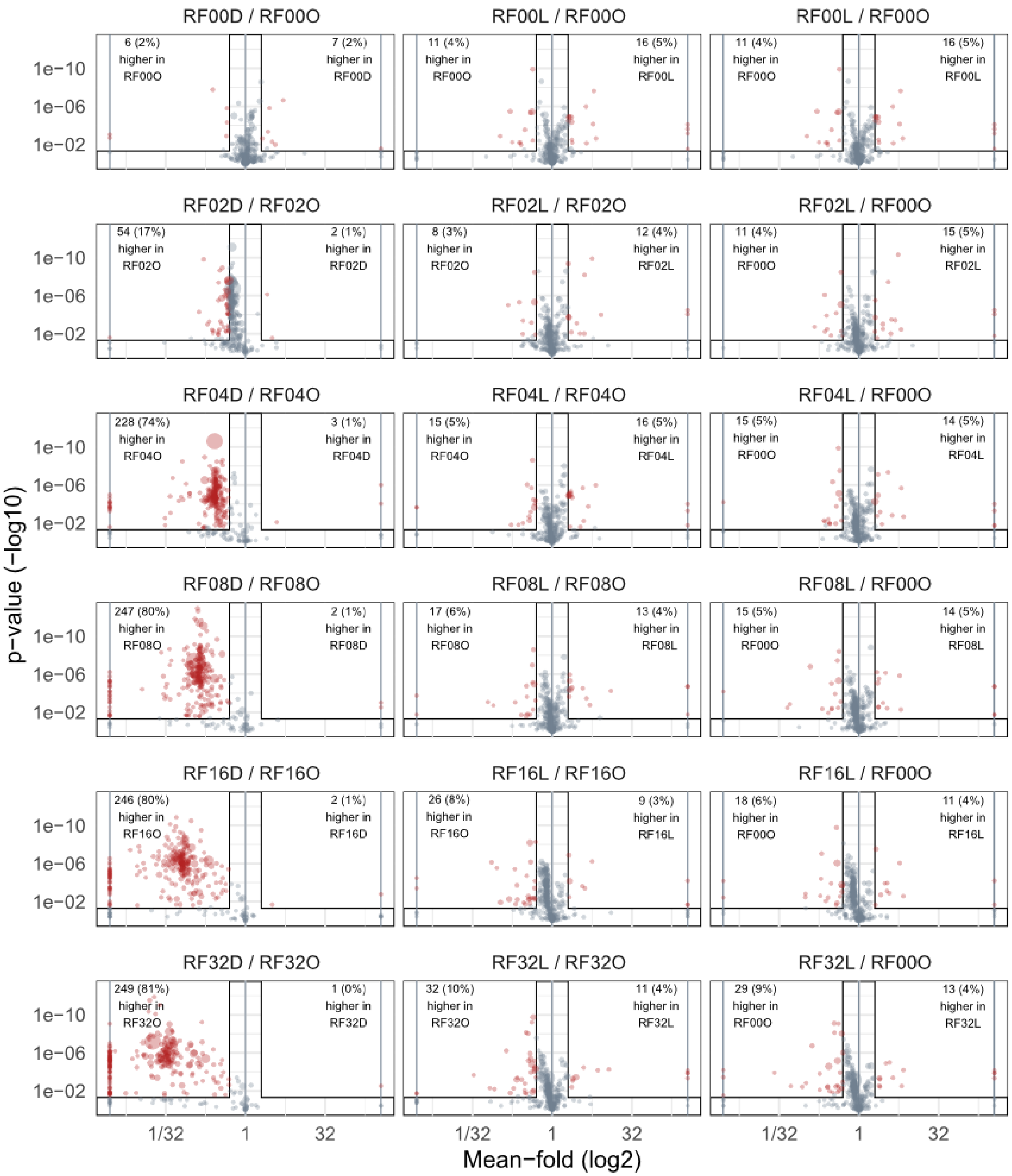

Figure S7. Overview of univariate tests of extracts of fresh roots (RF) before lyophilization (O), after dilution (D), and the after lyophilization plus reconstitution (L) at different dilution factors (number in group name). Grey dots indicate non-significantly differing metabolite abundances, red dots indicate significantly differing metabolite abundances (p-value < 0.05, mean-fold at least 2 or less than 0.5). The plots in the first column compare the diluted to the original extracts for each of the tested dilution factors. The plots in the second column compare the diluted to the concentrated samples of the respective dilution factor. The plots in the third column compare the concentrated extracts of the respective dilution factor to the original extracts of dilution factor 0.

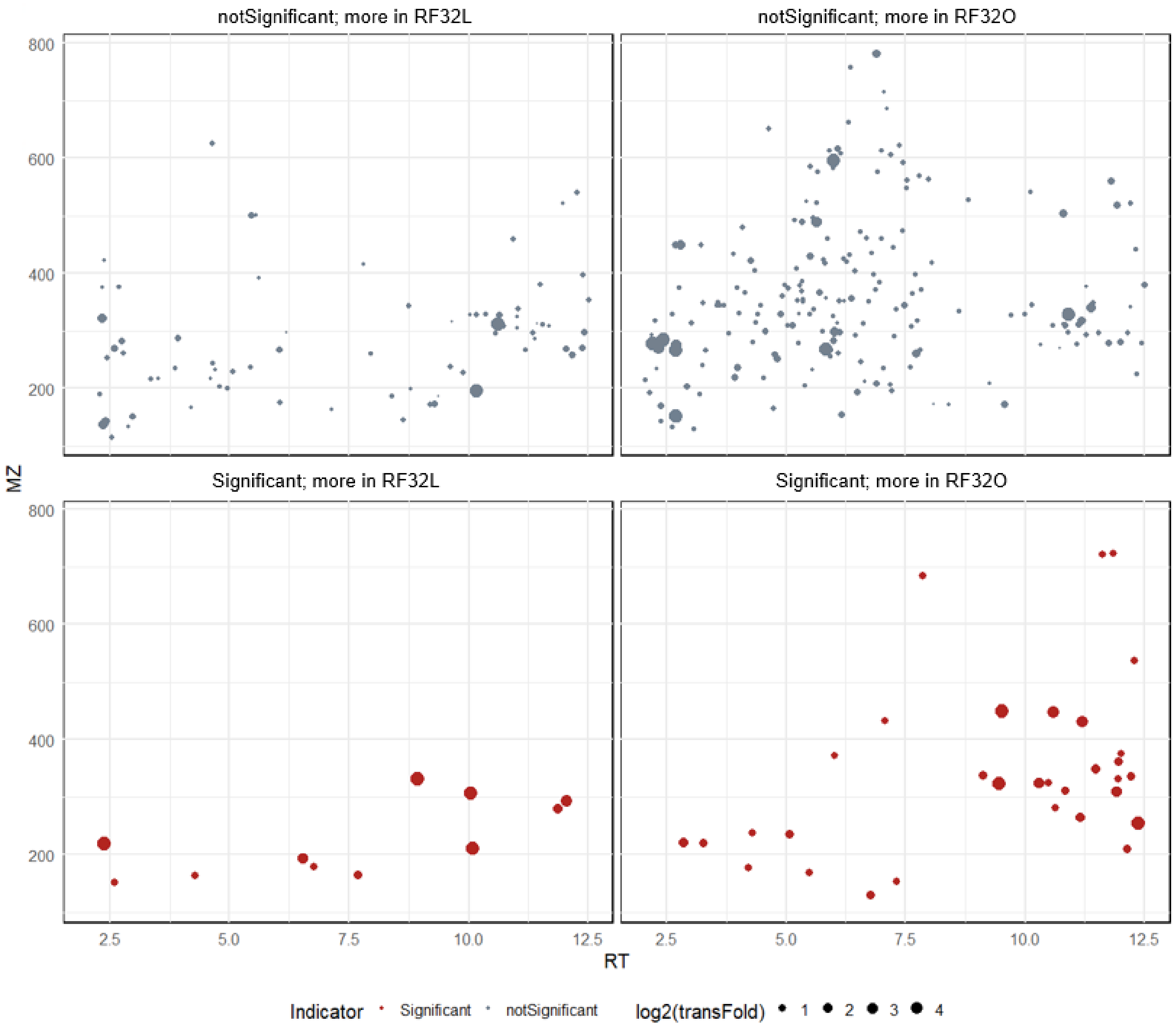

Figure S8. Feature plot (retention time versus *m*/*z* value) based on the results of the volcano plot (Figure S7) of the comparison of original and concentrated (lyophilized) extracts of fresh roots of factor 32. Dot size is proportional to peak area.

Experiment EC: Lyophilized roots

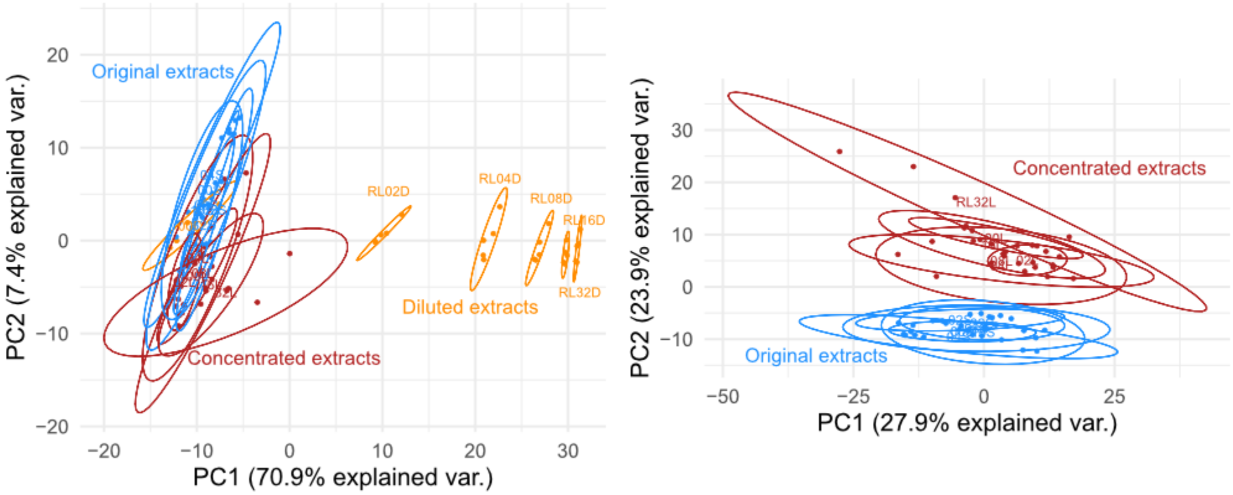

Figure S9. PCA scores plot of diluted (D, orange), lyophilized (L, red) and original (O, blue) samples obtained from lyophilized roots. The ellipses indicate the 95% confidence intervals for each group calculated from the respective replicates. Left: all samples and groups; Right: only the original and lyophilized samples.

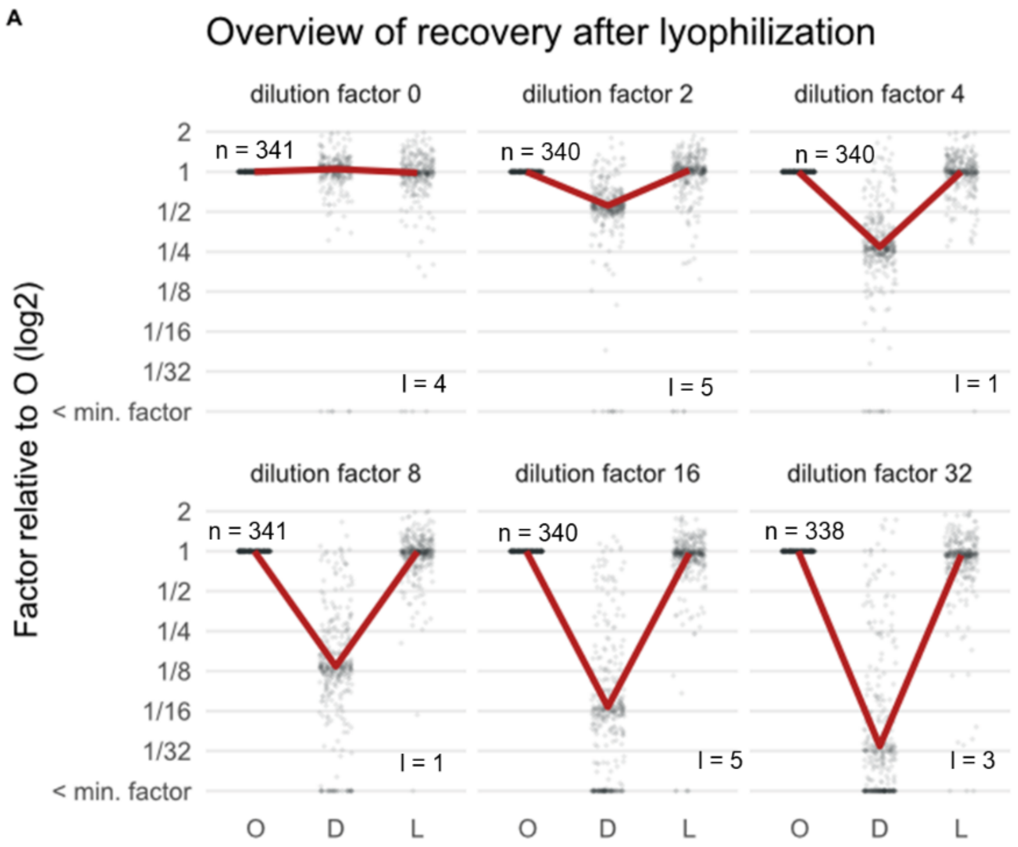

B

Type	Dilution factor	> min. factor	Median	Percentile 10%	Percentile 25%	Percentile 75%	Percentile 90%
L	1/0	314	0.98	0.71	0.91	1.12	1.36
L	1/2	311	1.03	0.68	0.94	1.13	1.36
L	1/4	316	1.00	0.66	0.92	1.13	1.34
L	1/8	328	0.99	0.69	0.91	1.10	1.30
L	1/16	320	0.96	0.69	0.86	1.05	1.26
L	1/32	319	0.94	0.62	0.81	1.02	1.29
O	1/0	341	1.00	1.00	1.00	1.00	1.00

Figure S10. A: Overview of metabolite recovery of extracts of dried roots before lyophilization (O), after dilution (factors 0 to 32) (D), and the recovery of this factor after concentrating by lyophilization (L). n indicates the number of metabolites detected in O, while l indicates the number of metabolites that have no longer been detected in L. The red line shows the mean value of all metabolites and the dots represent metabolites. B: Overview of how many metabolites were detected (> min. factor) in the respective standard mix, median and 10-, 25-, 75-, and 90-percentiles.

Data Processing parameters

Supporting Information S1. Parameters for MetExtract II .

Parameter	Parameter value
isotopic purity native and U-^{13}C-labeled material	98.87% and 99.50%
maximum allowed isotopolog deviation	+/- 15%
maximum allowed mass deviation	+/-3 ppm
scales width	3 – 19
minimum chromatographic peak correlation	0.85
retention time start and end	2 and 13 min
intensity threshold	1E3 units
EIC ppm	+/- 5 ppm
Cn counts to search for	3 – 60
retention time alignment	no
maximum bracketing deviation	8 ppm and 0.1 minutes

Supporting Information S2. Parameters for XCMS processing.

Parameter	Parameter value
method for peak picking	CentWave
chromatographic peak width	4 – 20 s
Pre-filter	3 x 1E5 intensity
signal-to-noise-threshold	3
CentWave maximum mass deviation	5 ppm

Supporting Information S3. Statistical analysis procedures.

Statistical analysis was carried out in R (https://r-project.org, version 3.5.3).
Only the peak areas of the native, monoisotopic compound forms were used. A feature must have been detected in at least 3 replicates of at least one experimental group in order to have been used and only the most abundant feature (ion) per metabolite was used. Missing values were replaced by zero. The data matrix was auto-scaled for multivariate analysis.
For Venn diagrams, a feature must have been detected in at least 3 replicates in order to have been assigned to the respective group.
Principal component analysis was done with the ggbiplot package (https://github.com/vqv/ggbiplot).
Heatmap and hierarchical cluster analysis (HCA) analysis was calculated using Euclidean distance and ward-linkage.
For Experiment EC Extraction Efficiency plots were generated showing an overview of metabolite recovery of samples before lyophilization, after dilution, and the recovery of this factor after lyophilization.
The Welch Two Sample t-test in combination with a mean-fold-change was used to test for a significant difference. A critical alpha value of 0.05 and a minimum mean-fold change of ≥ 2 or ≤ 0.5 formed significance threshold. For features only present in one group the mean-fold change is equal to 0 or an infinite value. Minimum Hedges G for effect size testing was set to at least 1.8.
